# Exploring the intergenerational persistence of health behaviour: an empirical study of smoking from China

**DOI:** 10.1186/s12889-017-4480-8

**Published:** 2017-06-08

**Authors:** Jay Pan, Wei Han

**Affiliations:** 1 West China School of Public Health, Sichuan University, No. 17, Section 3, Ren Min Nan Road, Chengdu, China; 2 West China Research Center for Rural Health Development Sichuan University, Chengdu, China; 3 Health, Nutrition and Population Global Practice, World Bank, 16F China World Office 2, No. 1 Jian Guo Men, Wai Avenue, Beijing, China

**Keywords:** Smoking, Intergenerational transmission, Health behaviour, China, I14, I12

## Abstract

**Background:**

It is of significance to look into the intergenerational transmission of risk behaviour to explain the disparity of health. Our paper contributes to the literature by providing evidence in the context of China, focusing on smoking behaviour.

**Methods:**

This paper studies the intergenerational transmission of smoking in the context of China using a nationally representative dataset – the China Health and Nutrition Survey (CHNS). The two-part model, the Tobit model, and the fixed effects model are utilized for the empirical analysis, respectively.

**Results:**

We found a strong intergenerational persistence of health behaviour. That is, parents’ smoking behaviour is positively correlated with their children’s smoking initiation.

**Conclusions:**

Our study provides evidence of the intergenerational persistence of health behaviour in the case of smoking, in the world’s most populous country. This has policy implications for the issue of intergenerational mobility and health education, as well as for tobacco control in China.

## Background

Social mobility, widely used as a measure of the equality of life opportunities, reflects the extent to which individuals can achieve success by virtue of their own talents and motivation, or by luck. Although China has experienced a remarkable increase in social mobility since its market-oriented economic reform in 1978 [1], this has tended towards stagnation over the last 10 years [2, 3].

A low degree of social mobility, or in other words high intergenerational persistence, means that parents have a significant influence on the outcome of their children’s later lives. Therefore, it is of importance to understand the mechanism of intergenerational transmission, in order to determine the optimum level of social mobility and whether government policy can play a role in achieving the optimum.

One mechanism is related to the investment in health, which is considered a fundamental dimension of human capital [4, 5], and the complementary relationships between health and other types of human capital investment such as education have received lots of attention [6]. Case et al. find that controlling for parental socioeconomic status, children suffering from poor health have significantly lower educational attainment, poorer health in their adulthood, and lower socioeconomic status, suggesting that health potentially plays a significant role in social mobility [7]. If a difference in human capital investment does exist, it can be expected to observe some degree of intergenerational persistence in a well-functioning market economy; however, the situation could be improved through government intervention to equalise opportunities [8].

Despite the fact that research into social mobility via the mechanism of human capital investment in health has been progressing rapidly, there are limited empirical studies devoted to risk behaviour, which accounts for more than 60% of the causes of death [9, 10]. Therefore, it is of significance to look into the intergenerational transmission of risk behaviour to explain health disparities. This paper contributes to the literature by providing evidence in the context of China, focusing on smoking behaviour.

Smoking is the leading preventable cause of death around the world, which is linked to an average of 1.2 million deaths annually in China [11, 12]. With the largest number of smokers in the world, China accounts for 40% of global tobacco production and consumption [11]. Given the fact that most smoking initiation in China occurs between 10 and 15 years old, preventing tobacco use among adolescents is essential to tobacco control, which could lead to a reduction in the public health burden from smoking-related illnesses during individuals’ lifetimes [13]. There has been an increasing trend with regards to the prevalence of smoking among adolescents over the past three decades, mostly caused by the progressive increase among females. While the estimated prevalence of lifetime smoking ranged between 39% and 46% for adolescent males in the period 1981–2010, it rose steadily from 2% to 19% for adolescent females during the same period [14]. In additional to the health hazards, adolescent smoking is highly associated with low learning productivity, as well as the number of other health risk behaviours in which these young adolescents had engaged [15, 16]. Parental smoking might be linked with adolescent smoking through many channels. For instance, children tend to imitate their parents’ behaviour, they may be tempted by exposure to second hand smoke, and parents and their children share similar time and risk preference [17, 18].

This paper studies the intergenerational transmission of smoking in the context of China using a nationally representative dataset – the China Health and Nutrition Survey (CHNS). The main outcomes of the essay relate to smoking initiation and tobacco consumption, therefore it contributes to the growing literature on this topic, and grounds evidence-based policy making on the issue of intergenerational mobility in China.

The paper is organised as follows. Section 2 briefly summarises the literature around the intergenerational transmission of health and risk behaviour, with a focus on smoking. Section 3 describes the data sources and the empirical methodology. Section 4 presents the findings, with section 5 offering conclusions.

**Literature review**

There is sizable literature studying the intergenerational transmission of smoking, but findings across studies are mixed [19]. Some studies report positive associations between parent and adolescent smoking, but not others. Even for the former, there was no consistency on whether or not the associations varied by gender (see Kandel et al. (2015) for a summary of relevant literature [20]).

There are some possible explanations for these mixed findings. Firstly, the published studies did not attempt to explore the causal relationship between parents’ and adolescents’ smoking behaviour, except for two notable studies that employed the instrumental variable method to overcome the unobserved family factors, such as risk attitudes and time preference. Nevertheless, those two studies did not reach a consistent conclusion. Loureiro et al. was the first study attempting to explore causality, which used social class and occupational indicators for the children’s grandparents as the instruments [17]. They found evidence of same-sex role models in two-parent households. Lillard used the price of cigarettes, and the amount of articles published about the health risks of smoking, as the instruments of the parents’ smoking status, and did not find any statistically significant evidence that the likelihood of children starting smoking depended on the former or current smoking status of their parents [19]. It is worth noting that Lillard’s paper explicitly pointed out that failing to address the endogeneity of parents’ smoking behaviour might lead to incorrect inferences. Indeed, completely different findings were reported in [21], which employed the same dataset as Lillard [19], but did not control for the endogeneity.

Secondly, estimations of the relationship between parent and adolescent smoking have suffered from the omitted variable problem, which potentially biases the estimation of the relationship [22]. Loureiro et al. [17] and Lillard [19] made an effort to deal with the endogeneity issue using the instrumental variable method, and, supposing the instruments they employed were fully valid (which is doubtful), what they achieved was to disentangle the link between children’s and their parents’ smoking habits from the presence of unobserved factors shared by all family members. However, the omitted variable problem was still not resolved. For instance, it has been well documented in the literature that the peer effect, i.e. the influence on their smoking behaviour of their schoolmates, friends, siblings, partners, and their neighbourhood, serves as an important driver of an individual’s smoking participation (see Christopoulou et al. (2013) for the summary of relevant literature [23]). Furthermore, some literature has implied that parenting style might be a factor in adolescents’ smoking behaviour [24–27].

Lastly, there is no consensus on how to measure variables of interest (i.e. parents’ and adolescents’ smoking behaviour) in order to derive valid policy implications from academic research. Adolescents’ smoking behaviour was mostly measured by smoking initiation [20, 27, 28], and/or whether an individual has smoked during the last week or month [13, 17, 27, 29]. Parents’ smoking behaviour was commonly measured by whether he or she was a current smoker [13, 17, 20, 27, 28], or a former smoker [20, 28]. As a result, it is difficult to understand the extent to which a wide variety of findings were caused by the measures of smoking status used in the above analyses.

There are some studies on the intergenerational transmission of smoking in China. In general, they reported that parental smoking was a significant risk factor for adolescents also smoking [13, 27, 30]. Nevertheless, there were some exceptions. Ma et al. found that adolescent smoking was strongly associated with peer smoking and low refusal self-efficacy, rather than parental smoking [31]. To the best of our knowledge, all of the published studies used self-completed questionnaires administered to a small number of pre-selected schools in a certain city, which has limited nationwide policy implications. The aim of this paper is to bridge the gap in the literature by studying the intergenerational transmission of smoking in the context of China using a nationally representative dataset.

## Methods

### Statistical analysis

Following the literature, a two-part model (2 PM) was used to characterise intergenerational smoking behaviour, which can be expressed as follows:

1 $$ \mathit{\Pr}\left({ASmoker}_i=1|{MSmoker}_i, FSmoker,{X}_i\right)=\Phi \left(\theta {MSmoker}_i+\delta FSmoker+{X}_i\eta +{\mu}_i\right) $$

2 $$ Ln\left({ANum}_i|{ASmoker}_i=1,{MSmoker}_i, FSmoker,{X}_i\right)=\chi {MSmoker}_i+\varphi FSmoker+{X}_i\gamma +{\upsilon}_i $$

where *ASmoker*_*i*_ and *ANum*_*i*_ are the binary variables indicating smoking status and the number of cigarettes smoked per day for adolescent *i*, respectively. *MSmoker*_*i*_ and *FSmoker*_*i*_,the key explanatory variables, denote whether adolescent *i*’s mother or father is a current smoker; the parameters, *θ*, *δ*, *χ*, and *φ*, are the coefficients of interest, representing the intergenerational effects of parental smoking on the probability of adolescent smoking, and the cigarettes consumption conditional on smoking (smoking intensity), respectively. *X*_*i*_ is a set of control variables, including the adolescents’ age, gender, and residential type (urban / rural), the parents’ age, years of education, employment status (not currently working, employed in collective, private, government, or other sectors), the annual household income per capita (measured in 2009 *Yuan*), and dummy variables identifying the survey years (1993, 1997, 2000, 2004, 2006, 2009) and residential regions (east, central, west).

The above 2 PM assumes that the smoking behaviour of adolescents is determined by two separate decision making processes: eq. (1), the “participation equation”, captures the systematic difference between smokers and non-smokers; eq. (2), the “intensity equation”, characterises the determination mechanism of the amount of cigarettes smoked among smokers, with the logarithm transformation on *ANum*_*i*_ used to reduce the impact of extreme values. Following the suggestion of previous studies [32], we estimate eq. (1) with the Logitistic model (specifying Φ(•) as the cumulative distribution function of the logistic distribution), and estimate eq. (2) with the Gamma GLM model (generalized linear model with a Gamma distribution for *ν*_*i*_). The specification is justified by the modified Park test, which shows that the conditional variance function of the distribution of the number of cigarettes smoked is consistent with the Gamma-class model. In addition, the Hosmer-Lemeshow test also confirms that our choice of log link function is consistent with the data generating process.

We also estimate the standard Tobit model for the purpose of checking robustness. As reviewed by [33], the 2 PM is frequently used in the literature on smoking behaviour because of the flexibility in the model specification. Specifically, the 2 PM assumes that the decisions to first start smoking and then the amount of cigarettes to consume are separately made. As a result, the regressions for the two parts (participation and intensity) can be separately estimated, bringing about the flexibility in the model specification. However, smoking participation and intensity might be a joint decision-making process rather than separate decisions. In the case of joint decision-making, the estimation from the Tobit model would be consistent, but the estimation from the 2 PM model would not from an econometric point of view. Given the fact that the two models each have their pros and cons, both will be estimated, and the results compared.

Data

Data from the China Health and Nutrition Survey (CHNS) is employed. The survey, an ongoing project since 1989, is conducted by the Carolina Population Centre at the University of North Carolina at Chapel Hill, in collaboration with the Chinese Centre for Disease Control and Prevention. Around 4400 households with a total of 26,000 individuals were sampled from 9 provinces (Guangxi, Guizhou, Henan, Heilongjiang, Hubei, Hunan, Jiangsu, Liaoning and, Shandong) using a multistage, random cluster process, which covers a wide range of geographical locations, economic development, social resources, and health utilisation. This longitudinal survey was conducted in 1989, 1991, 1993, 1997, 2000, 2004, 2006 and 2009. The CHNS is designed to examine the effects of the health, nutrition, and family planning policies implemented by the national and local governments, and to understand how the social and economic transformation affects the population’s health. It does this by collecting information on economics, health, family planning facilities, household nutrition and other social services, and community leaders.

The baseline data is excluded for this paper, since only the individuals aged between 20 and 45 were surveyed in 1989. For the present study, the data from 1991 to 2009 has been pooled, with the parent-child identifier used to filter a sample consisting of parent-offspring pairs. For families with multiple children, the family bond (and thus the family fixed effects) is identified by household IDs. The demographic, socio-economic and health-related information of the children and their parents is extracted from the CHNS child and adult questionnaires, respectively. Family income is extracted from the household questionnaire. Finally, our sample is restricted to adolescents aged between 13 and 18 following the literatures [30, 34]. Our study sample consists of 4368 adolescents with matching information for their parents. Table 1 presents details of the sampling procedure.

**Table 1:** Number of observations

Data	Number excluded	Number remaining
CHNS 1991, 1993, 1997, 2000, 2004, 2006, 2009		94,812
Restrict to observations with age 13–18	89,871	4941
Exclude if smoking status is missing	408	4533
Exclude if mother or father’s age is missing	10	4523
Exclude if household income is missing	27	4496
Exclude if mother or father’s education is missing	128	4368

The outcome of this study explains adolescent smoking behaviour, specifically regarding their smoking initiation and intensity. The former is measured by a binary variable which equals 1 if an individual is a smoker, and 0 otherwise. The latter is measured by the number of cigarettes smoked per day. The key explanatory variables, relating to parental smoking behaviour, are captured by the mother’s and father’s smoking status or their smoking intensity.

## Results

The descriptive statistics of key variables are reported in Table 2. Columns (1)–(3) represent the full sample, smoking sample, and non-smoking sample, respectively. As shown by Table 2, the prevalence of adolescent smoking is 3%, and most of them are males. Among the adolescent smokers, the average number of cigarettes consumed per day is approximately 8. In terms of parental smoking behaviour, more than 70% of adolescents’ fathers are smokers, who consume an average of around 15 cigarettes per day. On the contrary, only 2.5% of their mothers smoke, who, on average, smoke less than one cigarette per day.

**Table 2:** Descriptive statistics of variables

Variables	Total sample (1)	Smoking sample (2)	Non-smoking sample (3)
Smoking (=1)	0.030	1.000	0.000
Number of cigarettes smoked per day	0.254	8.346	-
Mother smoker (=1)	0.025	0.053	0.024***
Father smoker (=1)	0.715	0.902	0.709***
Number of cigarettes mother smoked per day	0.226	0.511	0.217*
Number of cigarettes father smoked per day	11.665	14.541	11.574***
Age	15.508	16.784	15.468***
Female (=1)	0.484	0.015	0.499***
Living in rural area (=1)	0.714	0.722	0.714
Mother’s age	42.237	43.679	42.191***
Father’s age	43.988	46.189	43.919***
Father’s education year	7.640	6.391	7.679***
Mother’s education year	5.729	4.887	5.755***
*Mother’s employment type*			
Unemployed	0.171	0.165	0.171
Farmer	0.113	0.143	0.112
Collective owned enterprises	0.470	0.519	0.468
Private or foreign enterprises	0.063	0.053	0.063
Government or state owned	0.160	0.075	0.162***
Other employment	0.010	0.015	0.010
*Father’s employment type*			
Unemployed	0.083	0.113	0.082
Farmer	0.103	0.135	0.102
Collective owned enterprises	0.432	0.459	0.431
Private or foreign enterprises	0.103	0.090	0.103
Government or state owned enterprises	0.253	0.135	0.256***
Other employment	0.011	0.023	0.011
Annual household income per capita	4464.620	4089.133	4476.412
*N*	4368	133	4235

Note: (1) The statistics reported are the sample mean. (2) Asterisks (***) denote statistically significant difference between the urban and rural groups (at 5% level). (3) The annual household per capita income is measured in 2009 *yuan* and is calculated by dividing the total household income by number of people in the family (parents and adolescents)

Table 3 reports the main results estimated by the 2 PM. Columns (1) and (2) are the results of the first and second part, respectively. The marginal effects are presented, with the standard errors clustered at the household level.

**Table 3:** Two part model results for adolescent smoking

Variables	Smoking (=1)	Number of cigarettes smoked per day
	(1)	(2)
Mother smoker (=1)	0.022**	0.257
	(0.010)	(0.266)
Father smoker (=1)	0.026***	0.432
	(0.007)	(0.263)
Age	0.124**	−4.928***
	(0.061)	(1.861)
Age square	−0.003*	0.151***
	(0.002)	(0.057)
Female (=1)	−0.091***	0.051
	(0.016)	(0.408)
Living in rural area (=1)	0.007	−0.148
	(0.006)	(0.219)
Mother’s age	−0.001	0.001
	(0.001)	(0.030)
Father’s age	0.001	−0.016
	(0.001)	(0.022)
Mother’s education year	0.000	0.006
	(0.001)	(0.030)
Father’s education year	−0.002**	0.000
	(0.001)	(0.023)
Mother’s employment type: Farmer	0.009	−0.929***
	(0.014)	(0.277)
Collective owned enterprises	0.008	−0.142
	(0.010)	(0.381)
Private or foreign enterprises	0.001	−0.439*
	(0.012)	(0.224)
Government or state owned	−0.001	−1.015**
	(0.013)	(0.421)
Other employment	0.027	−0.095
	(0.022)	(0.260)
Father’s employment type: Farmer	−0.003	0.775***
	(0.014)	(0.270)
Collective owned enterprises	−0.014	−0.287
	(0.010)	(0.221)
Private or foreign enterprises	−0.009	−0.268
	(0.012)	(0.258)
Government or state owned enterprises	−0.011	−0.071
	(0.011)	(0.223)
Other employment	0.001	0.734**
	(0.022)	(0.353)
Annual household income per capita	0.000	0.000
	(0.000)	(0.000)
Middle region	−0.017***	−0.037
	(0.005)	(0.171)
East region	−0.023***	−0.279
	(0.007)	(0.304)
Year Dummies	Yes	Yes
N	4368	133

Notes: (1) ***, ** and * denote statistical significance at 1%, 5% and 10% level, respectively. (2) The reported statistics are marginal effects, with clustered standard errors (at the household level) shown in parentheses

The baseline model shows that having a smoking mother and father significantly increased the probability of adolescents smoking by 2.2% and 2.6%, respectively, and this also had positive (albeit statistically insignificant) impacts on the smoking intensity (cigarette consumption) of their children who are smokers. The results suggest a strong intergenerational persistence of health behaviour; in other words, risky behaviour by the parents positively correlated with their children’s decision to engage in the same activity.

In terms of the other control variables, age had an inverted U-shaped effect on the likelihood of admitting to smoking, but a U-shaped impact on the number of cigarettes smoked among the smokers. That is, broadly speaking, the probability of adolescents engaging in risk behaviour escalates as they are growing up, but then declines after a certain age. Conversely, the intensity of risk behaviour had an opposite trend conditional on risk behaviour engagement. Female adolescents were less likely to smoke, but there was no significant gender difference in smoking intensity. The employment status of the parents seems to be linked with the amount of cigarettes consumed. Furthermore, compared to adolescents living in western regions, those who were living in middle and eastern regions are 1.7% and 2.3%less likely to admit to smoking, respectively. Lastly, neither household income nor whether or not an individual lives in a rural area had a significant impact on the probability of smoking initiation or cigarette consumption, respectively.

Where the 2 PM assumes that smoking initiation and smoking intensity are independent decisions, the Tobit model considers them a joint decision, to be jointly modelled and estimated [33]. Table 4 presents the results of the Tobit model. Columns (1) and (2) show the estimation of the expected number of cigarettes smoked conditional on being a smoker, using the parental smoking status and parental smoking intensity as the proxies, respectively. The marginal effect is reported with the standard errors clustered at the household level.

**Table 4:** Tobit model estimates for adolescent smoking

Variables	Number of cigarettes smoked per day	
	(1)	(2)
Mother smoker (=1)	0.755*	
	(0.393)	
Father smoker (=1)	0.718***	
	(0.182)	
Number of cigarettes mother smoked per day		0.050**
		(0.025)
Number of cigarettes father smoked per day		0.020***
		(0.006)
N	4368	4368

Notes: (1) ***, ** and * denote statistical significance at 1%, 5% and 10% level, respectively. (2) The reported statistics are marginal effects, with clustered standard errors (at the household level) shown in parentheses. (3) The same covariates as the models reported in Table 2, which are the adolescents’ age, sex, and residential type, the parental age, educational year, employment status, the annual household income per capita, region dummies, and year dummies

As shown in Table 4, parental smoking behaviour significantly affected adolescents’ smoking behaviour. On average, the adolescents with a smoking mother and father consumed 0.755 and 0.718 more cigarettes per day, respectively. When using parental smoking intensity as the proxy, we found that the adolescent consumed 0.5 and 0.2 cigarettes more on average if their mother and father were smoking 10 cigarettes or more per day, respectively. Overall, the results are virtually similar to in the 2 PM.

It should be noticed that one caveat of the 2 PM and Tobit models is that they only reveal the correlation rather than a causal relationship between the adolescents’ smoking behaviour and parental smoking status. First, unobservable factors such as genetic formation can possibly lead parents and their children to admit to smoking behaviour. Second, the parental smoking behaviour might be caused by their children’s smoking status; that is, the estimation potentially suffers from the “reverse causality” problem. From a statistical point of view, ignoring these endogeneity problems can bias our estimation, either upward or downward. Therefore, we further employed the Fixed Effects (FE) model to help identify the relationship, which can eliminate the unobservable and time-constant factors.

Table 5 reports the results from the FE model. Due to the limitation of the FE method, only variables with sufficient variation across time can serve as valid outcome variables in the regression. Considering that the smoking status varies little across the study periods, smoking intensity is used to proxy both of the adolescents’ and their parents’ smoking behaviour.

**Table 5:** Fixed effects model estimates for cigarettes that adolescent smoked

Variables	Number of cigarettes smoked per day
Number of cigarettes mother smoked per day	0.159***
	(0.055)
Number of cigarettes father smoked per day	−0.003
	(0.008)
N	3598

Notes: (1) ***denotes statistical significance at 1%. (2) The reported statistics are marginal effects, with clustered standard errors (at the household level) shown in parentheses. (3) The same covariates as the models reported in Table 2, which are the adolescents’ age, sex, and residential type, the parental age, educational year, employment status, the annual household income per capita, region dummies, and year dummies

The coefficient of the number of cigarettes smoked by the adolescents’ mother is significant and positive, as we should expect, but the coefficient for the father is insignificant and has a very small negative value. The results still confirm our main findings, but further reveal the gender difference, i.e. the mother’s smoking intensity had more significant influence on her children than their father’s did. There are two conjectures of why we see different effects from the mother’s behaviour and the father’s behaviour. Firstly, the proportion of father smoking is far too high comparing to that of mother (71.5% and 2.5% for the fathers and mothers, respectively, in our sample). So it is highly possible that the children may get used to father smoking but not mother smoking. Secondly, comparing with fathers, generally, mothers spend more time with the children. Therefore, the children could be more affected by mothers’ behaviour rather than fathers’.

## Discussion

In this paper, we studied the intergenerational transmission of smoking in the context of China using a nationally representative dataset. We found that parental smoking behaviour positively correlated with children’s decision to take up smoking, as well as the amount of cigarettes they consumed. This finding is similar to that of [21], who found that parental smoking behaviour significantly increased the children’s risk of starting smoking in Germany. Specifically, compared to adolescents with non-smoker parents, adolescents with both parents smoking led to an approximately 4.8% increase in the probability of smoking in China. A comparison of the results estimated by various models reveals the robustness of our findings.

Previous literature found a strong correlation between parents and their offspring of a broad range of health outcomes, such as self-assessed health [7, 35], obesity [36, 37], mental health [38], chronic health conditions [38, 39], and height and weight [40]. Given that health behaviour is a key attribute of health, a positive correlation between parents’ and their children’s health behaviour would play a substantially important role in the intergenerational transmission of health. Our results support these literature and provide the underlying mechanism.

The stagnation of social mobility in China has become a major concern in recent years, and has even brought about tension between the privileged class and the rest of the population. Despite the fact that well-designed public policy can play a part in improving equality of opportunity, individuals are indispensable to increase intergenerational transmission, not only in those for which they have primary responsibility, such as health behaviour, but also in human capital and income.

Furthermore, we should attach more importance to health education, particularly to adolescents, regarding the addictive properties of tobacco, as well as other risk behaviours. The experience from developed countries clearly indicates a gap between awareness - smoking is hazardous to one’s health - and practice – they still smoke**,** since they tend to neglect health risks from smoking the ‘very next cigarette’ [17].

Lastly, the Chinese government needs to make more efforts on tobacco control. We highly recommend the World Health Organization (WHO) Framework Convention on Tobacco Control (FCTC) as a guideline both for the policy-making and implementation in China. Smoking not only causes health hazards but also potentially has a negative influence on other dimensions of human capital, such as educational achievement, resulting in a multidimensional welfare loss. Besides, it not only affects smokers themselves but also non-smokers around them, and even the next generations. More specific and tough legislation on this issue is urgently needed, given that a nationwide ban on smoking in some indoor public places was introduced in 2011 but amounted to little. Furthermore, various anti-smoking campaigns should be promoted, such as calling on teachers not to smoke in front of students, and parents not to smoke in front of children, which will protect children from involuntary exposure to second hand smoke as well as from the effect of negative role models.

Our study has the following limitations: first, we only studied the correlation between the adolescents’ smoking behaviour and parental smoking status. Although the FE model could eliminate the unobserved and time-constant factors, the endogeneity problem might not be fully resolved. We are aware that, potentially, this issue can be addressed using an instrumental variable approach; however, we were not able to find an appropriate instrument that is essential to the validity of the study findings. Second, our estimation potentially suffers from omitted variable bias since we had no control of the peer effect or the quality of family relationships [13] due to the data availability - this has been well-documented as a key driver of smoking participation. Third, we used self-reported smoking status as the outcome variables, which could be subject to measurement errors. The adolescents tended to “under-report” their smoking behaviour since the surveys were carried out in front of their parents, thus the smoking status data could potentially suffer from the classic error-in-variable problem. Fourth, the analysed datasets were collected between 1991 and 2009. Therefore, our findings would be interpreted as a time average correlation between parental smoking behaviour and adolescent smoking behaviour. However, the smoking behaviour might change during this relatively long period of time, thus, it will be interesting to examine the intergenerational persistence over time for the future study. Finally, it should be noticed that the 4.8% percent increase in the probability of smoking when both mother and father smoke assumes independent effects of the smoking of both parents. Since we did not include a dummy variable into the regressions to examine the interaction effect of having both parents smoking at the same time, our estimations only account the independent effects of the mothers’ and fathers’ smoking behaviour on their offspring. In fact, we tried to include a dummy indicating whether both parents were smokers into the regression, however, we failed to get the estimation due to the high correlation between mothers’ smoking status and whether both parents were smokers. Specifically, the correlation coefficient is 0.90 for the total sample (*n* = 4368), and 1.00 (perfect collinearity) for the adolescents smoker sample (*n* = 133).

Nevertheless, even if our findings might not be interpreted as rigorous causal effects, the robust and significant correlations we found can enrich evidence regarding the intergenerational persistence of health behaviour, in the case of smoking. Most importantly, this has policy implications for the issue of intergenerational mobility, health education, and tobacco control in China.

## Conclusion

Our study provides evidence of the intergenerational persistence of health behaviour in the case of smoking, in the world’s most populous country. This has policy implications for the issue of intergenerational mobility and health education, as well as for tobacco control in China.
